# EVALUATION OF THE DEMENTIA FRIENDLINESS OF AIR TRAVEL—A CANADIAN PILOT STUDY

**DOI:** 10.1093/geroni/igad104.1044

**Published:** 2023-12-21

**Authors:** Linda Garcia, Alexandra Chiareli, Annie Robitaille, Michael Mulvey, Valentina Primossi

**Affiliations:** University of Ottawa, Ottawa, Ontario, Canada; University of Ottawa, Ottawa, Ontario, Canada; University of Ottawa, Ottawa, Ontario, Canada; University of Ottawa, Ottawa, Ontario, Canada; University of Ottawa, Ottawa, Ontario, Canada

## Abstract

An earlier initiative involving various stakeholders in the air travel industry, disability advocates, researchers, and individuals living with dementia and their travel partners identified five key areas to reduce the challenges faced by travellers living with cognitive limitations: there was a need for information and awareness on the topic, a discussion on disclosure and identification, a readjustment of processes and spaces, a need for formal training and standardized processes across jurisdictions. Next, focussing on current and planned initiatives for reducing these obstacles, the research team determined what might be available to consumers living with dementia and their families at Canadian airports. This symposium presentation will focus on the design and benefits of a citizen science approach for addressing the identified obstacles and the accessibility of the stated airport initiatives through the lens of passengers with dementia and their travel partners. Moreover, a prototype tool, adapted from the citizen science tool used in other presentations in this symposium, that includes data from the findings of both the stakeholder group and the airport analyses symposium will be presented and discussed in terms of its appropriateness and usefulness to evaluate the dementia-friendliness of air travel.

